# Implementing Mobile Health–Enabled Integrated Care for Complex Chronic Patients: Intervention Effectiveness and Cost-Effectiveness Study

**DOI:** 10.2196/22135

**Published:** 2021-01-14

**Authors:** Jordi de Batlle, Mireia Massip, Eloisa Vargiu, Nuria Nadal, Araceli Fuentes, Marta Ortega Bravo, Felip Miralles, Ferran Barbé, Gerard Torres, CONNECARE-Lleida Group

**Affiliations:** 1 Group of Translational Research in Respiratory Medicine Institut de Recerca Biomedica de Lleida Lleida Spain; 2 Centro de Investigación Biomédica en Red de Enfermedades Respiratorias CIBERES Madrid Spain; 3 Digital Health unit Eurecat Centre Tecnòlogic de Catalunya Barcelona Spain; 4 Gerència Territorial de Barcelona Institut Català de la Salut Barcelona Spain; 5 Atenció Primària Àmbit Lleida Lleida Spain; 6 Research Support Unit Lleida Fundació Institut Universitari per a la recerca a l'Atenció Primària de Salut Jordi Gol i Gurina Lleida Spain; 7 Centre d'Atenció Primària Cappont Gerència Territorial de Lleida Institut Català de la Salut Lleida Spain; 8 Universitat de Lleida Lleida Spain; 9 Please see acknowledgements section for list of collaborators

**Keywords:** chronic disease, cost-benefit analysis, delivery of health care, integrated, mHealth, eHealth, quality of life

## Abstract

**Background:**

Integrated care can generate health and social care efficiencies through the defragmentation of care and adoption of patient-centered preventive models. eHealth can be a key enabling technology for integrated care.

**Objective:**

The aim of this study was to assess the effectiveness and cost-effectiveness of the implementation of a mobile health (mHealth)-enabled integrated care model for complex chronic patients.

**Methods:**

As part of the CONNECARE Horizon 2020 project, a prospective, pragmatic, two-arm, parallel implementation trial was held in a rural region of Catalonia, Spain. During 3 months, elderly patients with chronic obstructive pulmonary disease or heart failure and their carers experienced the combined benefits of the CONNECARE organizational integrated care model and the eHealth platform supporting it, consisting of a patient self-management app, a set of integrated sensors, and a web-based platform connecting professionals from different settings, or usual care. We assessed changes in health status with the 12-Item Short-Form Survey (SF-12), unplanned visits and admissions during a 6-month follow up, and the incremental cost-effectiveness ratio (ICER).

**Results:**

A total of 48 patients were included in the integrated care arm and 28 patients receiving usual care were included in the control arm (mean age 82 years, SD 7 years; mean Charlson index 7, SD 2). Integrated care patients showed a significant increase in the SF-12 physical domain with a mean change of +3.7 (SD 8.4) (*P*=.004) and total SF-12 score with a mean change of +5.8 (SD 12.8) (*P*=.003); however, the differences in differences between groups were not statistically significant. Integrated care patients had 57% less unplanned visits (*P*=.004) and 50% less hospital admissions related to their main chronic diseases (*P*=.32). The integrated care program generated savings in different cost scenarios and the ICER demonstrated the cost-effectiveness of the program.

**Conclusions:**

The implementation of a patient-centered mHealth-enabled integrated care model empowering the patient, and connecting primary, hospital, and social care professionals reduced unplanned contacts with the health system and health costs, and was cost-effective. These findings support the notion of system-wide cross-organizational care pathways supported by mHealth as a successful way to implement integrated care.

## Introduction

The last decades have led to rapidly aging populations and an increased burden of chronic diseases [1]. In this scenario, health and social care providers struggle to contain costs while providing an adequate response to the population’s care needs. Traditional care models suffer from care fragmentation. That is, the different care settings fail to communicate with each other effectively, and patients have to undergo repeated tests and changes in prescribed drugs, ultimately prompting feelings of starting anew after every transition [2]. Additionally, the focus is still on the diseases rather than on the patients, which leaves patients and their carers as passive actors [2]. Therefore, there is a need for a profound redesign of how care is provided to elderly patients with chronic conditions to ensure quality and sustainability [3]. Integrated care models aim to generate health and social care efficiencies through the defragmentation of care, promotion of collaboration and continuity of care across settings, adoption of patient-centered models, and prioritization of preventive models [4]. However, few of these models have attempted to and succeeded in simultaneously tackling all of the above-mentioned measures [4]. eHealth and mobile health (mHealth) can be the key enabling technologies allowing for such a paradigm shift [5].

The Personalised Connected Care for Complex Chronic Patients (CONNECARE) project is a Horizon 2020 European Union Research and Innovation project aiming to co-design, develop, deploy, and evaluate a novel smart and adaptive organizational integrated care model for complex chronic patients (CCP) [6]. From April 2016 to December 2019, the project successfully co-designed, by means of an iterative patient-centered process involving patients and stakeholders across different health settings, an organizational model for integrated care and an eHealth platform to support the model. The integrated care model promotes collaboration among professionals of different care settings (family physicians, hospital specialists, and social workers), prioritizes home-based prevention over institutional reactive care, and fosters patient empowerment. The use of a web-based platform offers a cross-setting web-based Smart Adaptive Case Management (SACM) system for professionals, and an mHealth self-management system with three-level monitoring features allowing for patient empowerment.

As part of the CONNECARE project, a novel mHealth-enabled integrated care model was implemented in Lleida, Spain. The existing care model in Lleida is limited by the scarce communication between professionals of different care settings, with different electronic medical record (EMR) systems in hospitals and primary care centers (Argos SAP and ECAP [7], respectively), and patients playing a passive role throughout the care path. We here describe the results of the implementation of an mHealth-enabled integrated care model for the community-based prevention of unplanned hospital-related events in CCP with a high risk for hospitalization.

## Methods

### Study Design

This was a prospective, pragmatic, two-arm, parallel implementation trial comparing care as usual to a 3-month mHealth-enabled integrated care intervention. The study was conducted from July 2018 to August 2019 in Lleida, which is a large rural area of over 4300 km^2^, including two tertiary hospitals (University Hospital Arnau de Vilanova and University Hospital Santa Maria) and a network of 23 primary care centers spread across the whole territory, providing service to 400,000 citizens.

### Target Population

Home-dwelling patients with chronic conditions and a history of hospitalizations were recruited for this study. The eligibility criteria were aged ≥55 years; admitted to hospital for a respiratory or cardiovascular event (ie, chronic obstructive pulmonary disease exacerbation or heart failure decompensation); living at home and discharged back to the community; no dementia or cognitive impairment (Global Deterioration Scale score < 5 [8]); Length, Acuity, Comorbidities and Emergency score > 7 [9]; and passing a basic technological test assessing home connectivity and patients’ or carers’ competences with the use of technology. The basic technological test is shown in Multimedia Appendix 1.

### Recruitment

Patients were recruited during an unanticipated admission to the hospital through the emergency room (ER). They were identified based on EMR data and were contacted by a case manager before discharge. After the recruitment of patients to the intervention arm, an active search for a matched control with similar characteristics began. All patients and their carers, regardless of study arm, received a face-to-face explanation about the study.

### Intervention

Patients in the intervention arm experienced an integrated care model, including (i) preliminary assessment of the patient’s health status using several questionnaires, tests, and indices specific to their main chronic diseases and social needs; (ii) a self-management app, with status and performance reports, a virtual coach with customizable automated feedback, and full communication with the care team; (iii) a Fitbit Flex 2 digital activity tracker [10] and any additional sensor deemed necessary by the care team [11], including a digital pulse-oximeter, digital scale, and digital blood pressure monitor, that were fully integrated into the self-management app; (iv) a patient profile in the SACM web-based platform, accessible to all members of the care team (family physicians, hospital specialists, and social workers), that was used for coordination and communication among professionals in the different settings, and to contact the patient when needed; and (v) assignment of a case manager in charge of supervising the whole process and serving as the main patient contact point. Additional details on the CONNECARE integrated care model and the supporting eHealth platform can be found in Multimedia Appendix 1. Patients in the control arm experienced care as usual, managed from primary care. After discharge from the initial 90 days of usual care for integrated care management, all patients were passively followed up for an additional 3 months.

### Data Collection

Variables characterizing the patients were collected at recruitment using the SACM in tablet or desktop computers, including age, sex, main chronic disease(s), Charlson index of comorbidities [12], quality of life (QoL) as measured by the 12-Item Short-Form Survey (SF-12) [13], Barthel index for Activities of Daily Living [14], Hospital Anxiety and Depression scale [15], assessment of dwelling characteristics, main medications, Pfeiffer mental status questionnaire [16], and tobacco and alcohol consumption. The main outcomes were: (i) intervention effectiveness, as measured by the changes in the SF-12 health questionnaire’s physical and mental domains (baseline vs discharge); (ii) use of health care resources after 6 months, and estimated associated costs based on Catalan Health Department official data [17]; and (iii) cost-effectiveness, based on the improvement in QoL relative to costs, assessed by means of the incremental cost-effectiveness ratio (ICER); all costs are described in US $ (conversion factor: 1 € = 1.21 US $). Additional details on cost estimations are described in Multimedia Appendix 1. The use of health care resources was collected from EMRs, which included hospital admissions, ER visits, visits to primary care, and visits to hospital specialists. Additionally, each admission or visit was assessed regarding its relation to the patient’s chronic diseases as a binary variable (related or unrelated).

### Statistical Analyses

Participants’ baseline characteristics are summarized as n (%), mean (SD), or median (IQR) as appropriate. Comparisons between baseline characteristics of patients in the integrated care and control groups were performed using the χ^2^ test, *t* test, or Kruskal-Wallis test, as appropriate. A paired *t* test was used to compare baseline to discharge values with respect to the SF-12 domains. Linear regression models were used to assess differences in the changes experienced by patients in the two groups. Negative binomial regression models were used to assess differences in the number of visits and admissions. Models were adjusted by age, sex, and Charlson comorbidity index. The ICER was calculated in relation to the SF-12 total score in three different scenarios: 100%, 150%, and 200% estimated cost of the integrated care program. Data analyses were conducted using Stata version 12.1 (StataCorp, College Station, TX, USA). The threshold for significance was P<.05.

### Ethical Considerations

This study was approved by Ethics Committee of Hospital Arnau de Vilanova (CEIC-1685) and all patients provided written informed consent. All collected data were handled and stored in accordance with current national and international legislation.

## Results

**Figure 1:**
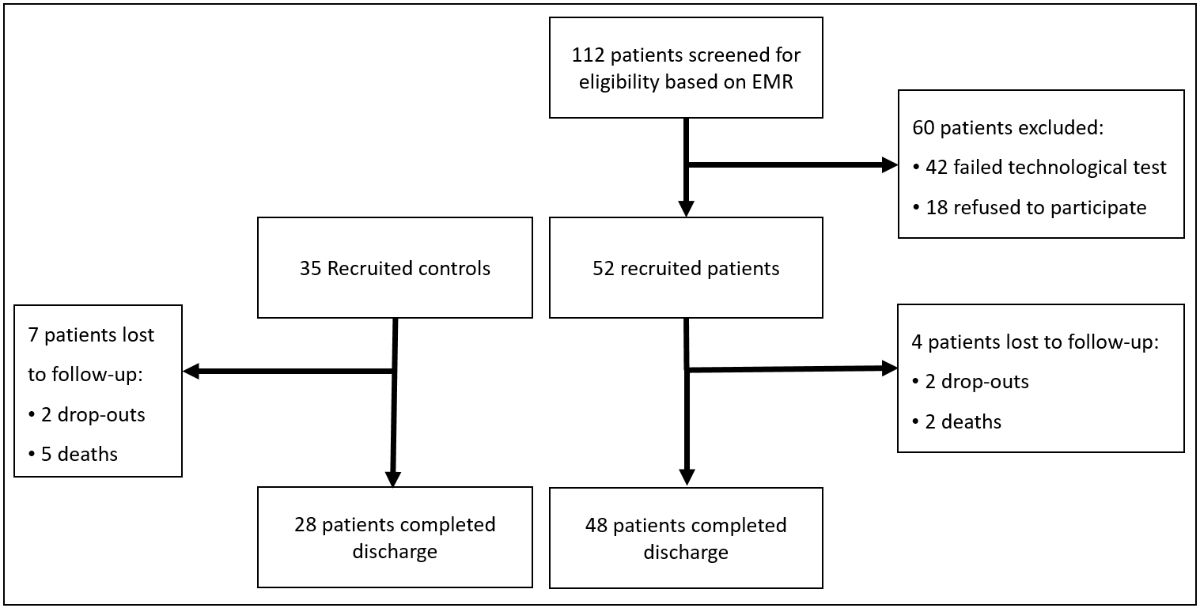
Study flowchart. EMR: electronic medical record.

Up to 112 patients were screened for eligibility. After excluding patients not meeting the inclusion criteria, 52 patients were recruited for the mHealth-enabled integrated care arm and 35 patients were recruited for the usual care arm. Final analyses were based on 48 integrated care and 28 control patients completing the follow up (Figure 1).

The patients’ baseline characteristics are shown in Table 1. There were no significant differences in mean age or Charlson index between the patients in the two arms.

**Table 1:** Baseline characteristics of patients in the usual care and integrated care (IC) arms.

Characteristic	Usual care (n=28)	IC (n=48)	*P* value^a^
Sex (male), n (%)	17 (61)	24 (50)	.37
Age (years), mean (SD)	82 (8)	82 (7)	.88
Charlson score, mean (SD)	7.4 (2.1)	6.7 (2.0)	.15
LACE^b^ score, median (IQR)	15 (13-17)	14 (12-17)	.38
Barthel score, median (IQR)	90 (72.5-95)	90 (67.5-100)	.40
HAD^c^ anxiety score, mean (SD)	4.9 (3.5)	4.3 (2.7)	.44
HAD depression score, mean (SD)	5.6 (2.9)	5.7 (2.3)	.95
Pfeiffer intact intellectual functioning, n (%)	21 (75)	37 (77)	.67
GDS^d^, no cognitive decline, n (%)	27 (96)	44 (92)	.25

^a^χ^2^ test, *t* test, or Kruskal-Wallis equality-of-populations rank test, as appropriate.

^b^LACE: Length, Acuity, Comorbidities, and Emergency score.

^c^HAD: Hospital Anxiety and Depression scale.

^d^GDS: Global Deterioration Scale.

Table 2 shows the changes in QoL (SF-12 domains) from baseline to discharge. Patients in the integrated care arm showed a significant increase in the SF-12 physical domain and total SF-12 scores. The differences in QoL between integrated care and control patients favored the integrated care patients but did not achieve statistical significance.

**Table 2:** Changes in health status in the usual care and integrated care arms.

SF-12^a^ score		Baseline, mean (SD)	Discharge, mean (SD)	Change, mean (SD)	*P* value^b^
**Physical**					
	Usual care	29.6 (8.3)	31.6 (9.0)	+2.0 (7.5)	.16
	Integrated care	29.0 (7.3)	32.7 (9.4)	+3.7 (8.4)	.004
	Difference	–0.6 (2.0)	+1.1 (2.0)	+1.7 (2.9)	.21
**Mental**					
	Usual care	47.0 (13.5)	45.8 (15.5)	–1.2 (11.9)	.59
	Integrated care	51.8 (9.9)	53.9 (11.5)	+2.0 (11.2)	.21
	Difference	+4.2 (3.7)	+9.2 (3.7)	+5.0 (5.2)	.10
**Total**					
	Usual care	76.6 (13.9)	77.4 (20.5)	+0.8 (14.7)	.77
	Integrated care	80.8 (11.9)	86.6 (16.3)	+5.8 (12.8)	.003
	Difference	+4.2 (3.7)	+9.2 (3.7)	+5.0 (5.2)	.10

^a^SF-12: 12-Item Short-Form Survey.

^b^Paired *t* test comparing baseline and discharge measures; linear regression predicting the difference on baseline and discharge measures according to intervention arm, adjusted by age, sex, and Charlson index.

Table 3 shows that integrated care patients had 57% less unplanned visits, representing a significant difference. Integrated care patients also experienced a 50% reduction in hospital admissions related to their main chronic diseases, although this difference was not statistically significant.

**Table 3:** Total use of health services during the follow-up period.

Health service	Usual care (n=28), mean (SD)	Integrated care (n=48), mean (SD)	*P* value^a^	Adjusted *P* value^b^
All unplanned visits	2.3 (3.1)	1.0 (1.1)	.001	.004
Unplanned visits related to chronic disease	0.9 (1.2)	0.4 (0.6)	.01	.04
All hospital admissions	0.5 (0.8)	0.4 (0.6)	.35	.50
Hospital admissions related to chronic disease	0.4 (0.7)	0.2 (0.5)	.18	.32

^a^Negative binomial regression model.

^b^Negative binomial regression model adjusted by age, sex, and Charlson comorbidity index.

The results for the within trial costs and cost-effectiveness analyses for all unplanned visits and hospital admissions are shown in Table 4, and those for unplanned visits and hospital admissions related to the patient’s main chronic diseases are shown in Table S1 of Multimedia Appendix 1. The integrated care program generated savings from US $584 to $1434 per patient, depending on the scenarios. The integrated care program was cost-effective according to the ICER, performing better in terms of QoL while reducing overall expenses.

**Table 4:** Within trial costs (average cost per patient) and cost-effectiveness considering all unplanned visits and hospital admissions in three integrated care (IC) program cost scenarios.

Related cost		Usual care (n=28), US $	IC (n=48), US $	Difference	ICER^a^
Unplanned visits^b^		173.62	78.02	–95.60	N/A^c^
Hospital admissions^b^		3069.94	2404.15	–665.79	N/A
Total medical costs per patient		3243.56	2482.17	–761.39	N/A
**Scenario 1: 100% IC program costs**					
	CONNECARE program	0	85.92	85.92	N/A
	Total costs per patient	3243.56	2568.09	–675.47	–135.64
**Scenario 2: 150% IC program costs**					
	CONNECARE program	0	128.89	128.89	N/A
	Total costs per patient	3243.56	2611.06	–632.50	–127.01
**Scenario 3: 200% IC program costs**					
	CONNECARE program	0	171.84	171.84	
	Total costs per patient	3243.56	2654.01	–589.55	–118.39

^a^ICER: incremental cost-effectiveness ratio; incremental cost associated with 1 additional point gain in 12-Item Short-Form Survey (SF-12).

^b^Costs based on the Catalan Institute of Health official pricing (CVE-DOGC-A-13051031-2013).

^c^N/A: not applicable.

## Discussion

### Principal Results

The prospective assessment of the implementation of an mHealth-enabled integrated care program for CCP management showed a reduction in the number of unplanned contacts with the health system, generated substantial savings for the health system without having any negative impact on QoL or clinical outcomes, and demonstrated cost-effectiveness.

### Strengths and Limitations

A key strength of this study was the effort to involve, from the very beginning, all of the stakeholders from different organizations that would be actors in a large-scale deployment of the mHealth-enabled integrated care program. This is especially relevant as the lack of cooperation between organizations, teams, or professions is a recurrent barrier toward the implementation of integrated care [4]. Other relevant strengths included: (i) the involvement of informal carers in the integrated care process, as close relatives of patients are usually the link between the patients and the health system, and such informal carers were key for facilitating the use of the self-management app in patients with a mean age over 80 years; (ii) the use of a self-management app (including a virtual coach with customizable automated feedback, full communication with the care team, and active monitoring), as such apps can significantly enhance doctor-patient relationships [18] and detect worsening in patient conditions or frailty [19]; (iii) the promotion and assessment of patients’ physical activity, as mobility impairment is found in one third of people over 65 years [20]; (iii) the implementation region, a large rural area of over 4300 km^2^, which can benefit the most from community-based integrated care initiatives that preclude unnecessary travels to the hospital; and (iv) the prospective study design.

In terms of limitations, although the organizational model did not experience substantial changes throughout the implementation period, the supporting technological platform was in a permanent process of refinement and addition of new functionalities. This implied that the integrated care experience was richer in patients who were recruited near the end of the implementation study compared with that of patients recruited at the very beginning. Similarly, this had a considerable impact on the health care professionals, who had to cope with a platform in constant development and that was not fully integrated with the existing EMR. However, directly participating in a dynamic development and implementation process allowed the professionals to feel engaged and propose changes, and for new features to be developed, which ultimately resulted in not a single professional dropping out of the implementation study. Other limitations were: (i) the relatively small number of patients involved in this first phase of the deployment, which ultimately affected statistical power; (ii) the assumptions held while determining the costs of the mHealth-enabled integrated care program intervention, as well as the lack of assessment of indirect (societal) savings; and (iii) the strategy of centralizing the entry points to the integrated care program in the hospital (after ER admission), as it is important that system-wide cross-organizational care pathways consider multiple entry points [21]. In this regard, upcoming phases of the implementation will consider additional entry points such as the primary care centers.

### Comparison With Existing Literature

The impact of the implementation of the integrated care model was assessed in three domains: (i) patients’ QoL, (ii) use of health services, and (iii) economics. In the first domain, QoL, the integrated care model performed slightly better than usual care, with positive differences-in-differences values but not reaching statistical significance. This result is in line with a 2017 umbrella review concluding that integrated care interventions showed mixed results in terms of improving patients’ QoL [22]. In this sense, it must be noted that the short duration of the implemented intervention (3 months) could have limited its capacity to affect the overall QoL. Nevertheless, the short duration did not preclude obtaining excellent results in terms of the use of health services. The integrated care model reduced the number of unplanned visits by 57% and the number of hospital admissions related to the main chronic disease of each patient by 50%. Half of the published reviews on integrated care interventions between 2000 and 2015 reported significant reductions in hospital activity, ranging from 15% to 50% [23]. This places the reported results for the integrated care program within the top margin of positive results, and supports the notion of system-wide cross-organizational care pathways as a successful way to implement integrated care in contrast to smaller and narrow interventions [2]. Finally, regarding the economic impact, integrated care generated savings from US $584 to $1434 per patient and was cost-effective. This is in line with reviews stating the potential cost-effectiveness of integrated care for the management of chronic diseases [24]. Our results are also in line with savings found in other chronic diseases such as diabetes mellitus (from –1508 to +299 Euro; approximately US $–1809 to +359) or schizophrenia (from –3860 to +614 Euro; approximately US $–4632 to 737) [25].

### Implications for Research and Practice

The need for people-centered integrated care, capable of providing an adequate response to the needs of growing populations of older people with chronic conditions while keeping costs sustainable, has been clearly stated by the World Health Organization [26]. In the frame of the CONNECARE project, an mHealth-enabled integrated care model was co-designed through an iterative process that involved all of the key stakeholders: patients, hospital and primary care medical and technical staff, social carers, managers, developers, and researchers. This coproduction multidisciplinary team had a clear focus on the patient and was ready to consider system-wide cross-organizational care pathways. The resulting mHealth-enabled integrated care model was thus perfectly aligned with the 2015 World Health Organization report on aging and health [1], which states that care providers should ensure that: (i) the assessment of individual impairments/declines in capacity is used to inform the development of a comprehensive care plan, and all domains are assessed together; (ii) interventions encouraging physical exercise are included in the care plans; and (iii) the presence of any impairment/decline in capacity triggers actions for the medical assessment of associated diseases. Moreover, we identified key features to be included in successful integrated care models for elder CCP: (i) the involvement of informal caregivers, being key in the adoption of mHealth tools such as self-management apps and sensors, and making the overall user experience very satisfactory [27]; (ii) the enablement of a common web-based platform for the coordination of care across settings and patient follow up; and (iii) the enhancement of communication channels for patients, which reduced the need of face-to-face appointments for quick consultations or questions. This latter aspect is especially relevant when patients are depending on others for travel to the general practitioners’ offices or primary care centers.

### Conclusion

The implementation of a patient-centered mHealth-enabled integrated care model empowering patients and connecting primary, hospital, and social care professionals reduced unplanned contacts with the health system and health costs, and was cost-effective. This supports the notion of system-wide cross-organizational care pathways using mHealth tools as a successful way to implement integrated care.

## Appendix

Multimedia Appendix 1 Online supplement.

## Abbreviations

CCP: complex chronic patients
CONNECARE: Personalised Connected Care for Complex Chronic Patients
EMR: electronic medical records
ER: emergency room
ICER: incremental cost-effectiveness ratio
mHealth: mobile health
QoL: quality of life
SACM: Smart Adaptive Case Management
SF-12: 12-Item Short-Form Survey
