# Supplementary material for: Implementing Mobile Health–Enabled Integrated Care for Complex Chronic Patients: Intervention Effectiveness and Cost-Effectiveness Study
Source: JMIR Mhealth Uhealth. 2021 Jan 14;9(1):e22135. doi: 10.2196/22135 (PMC7843204; doi:10.2196/22135)
Supplement: Multimedia Appendix 1 [file mhealth_v9i1e22135_app1.docx]

**SUPPLEMENTAL INFORMATION**

Implementing mHealth-enabled Integrated Care for Complex Chronic Patients: an intervention effectiveness and cost-effectiveness study

Jordi de Batlle, PhD, Mireia Massip, BSc(Nurs), Eloisa Vargiu, PhD, Nuria Nadal, MD, Araceli Fuentes, MD, Marta Ortega Bravo, MD, Felip Miralles, PhD, Ferran Barbé, MD, Gerard Torres, MD, on behalf of the CONNECARE-Lleida group.

Contents

[Basic technological test for patients and carers 2](#_Toc49329483)

[The Integrated Care model 2](#_Toc49329484)

[Technical structure of the supporting eHealth platform 8](#_Toc49329485)

[Implementation efforts 9](#_Toc49329486)

[Costs estimations 9](#_Toc49329487)

[References 10](#_Toc49329488)

[Supplementary tables 11](#_Toc49329489)

# Basic technological test for patients and carers

As eligibility criteria, patients and/or carers had to pass a basic technological test assessing home connectivity and patients and/or carers’ competences with the use of technology. The test consisted on the following 3 items:

1. Do you or your caregiver have an Internet connection?

- Yes
- No

1. Are you confident using:

- Smartphone
- Tablet computer
- Personal computer
- None of the above

1. Is your main carer confident using:

- Smartphone
- Tablet computer
- Personal computer
- None of the above

[This short test was considered as successfully passed if the patient and/or caregiver reported having an internet connection and being confident in the utilization of at least one of the proposed devices]

# The Integrated Care model

The Integrated Care (IC) model was based on a 5-step strategy: Case Identification, to select patients eligible for entering the program; Case Evaluation, to stratify patients based on clinical, environmental and social risk assessments; Work-plan definition, to plan personalized proactive and preventive interventions; Work-plan execution, to continuously monitor the evolution of the personalized care plan (through questionnaires, measurements with medical devices, educative material, and bidirectional communication wrapped-up in a patient self-management App); and, Discharge, to evaluate the overall intervention. The five steps were accommodated into a Smart Adaptive Case Management (SACM) web-based platform accessible to all involved professionals in the different settings (family physicians, hospital specialists and social workers), that guided professionals while keeping record of all relevant data. A detailed description of each of these steps as well as the technological tools that were used is presented below.

1. Case Identification

Potential patients to be included in the program were identified, based on electronic medical records (EMR) data. A nurse case manager contacted candidates before their discharge of an unanticipated admission to the hospital through the emergency room (ER). The case manager duties included the presentation of the study to the candidates, the collection of the patient’s informed consent form, the generation of a patient’s profile in the SACM platform, and the assessment of inclusion/exclusion criteria using such platform. Figure S1 shows the main screen of the SACM for the case manager. Figure S2 shows how the platform guides the case manager through the required steps for patient inclusion. At the time of new case generation, the case manager assigned the multidisciplinary team of professionals that would collaborate in the management of the patient, which could then be modified at any time. This first contact with the patient was usually performed in the patient’s hospital room using a tablet computer, which allowed for the required flexibility.


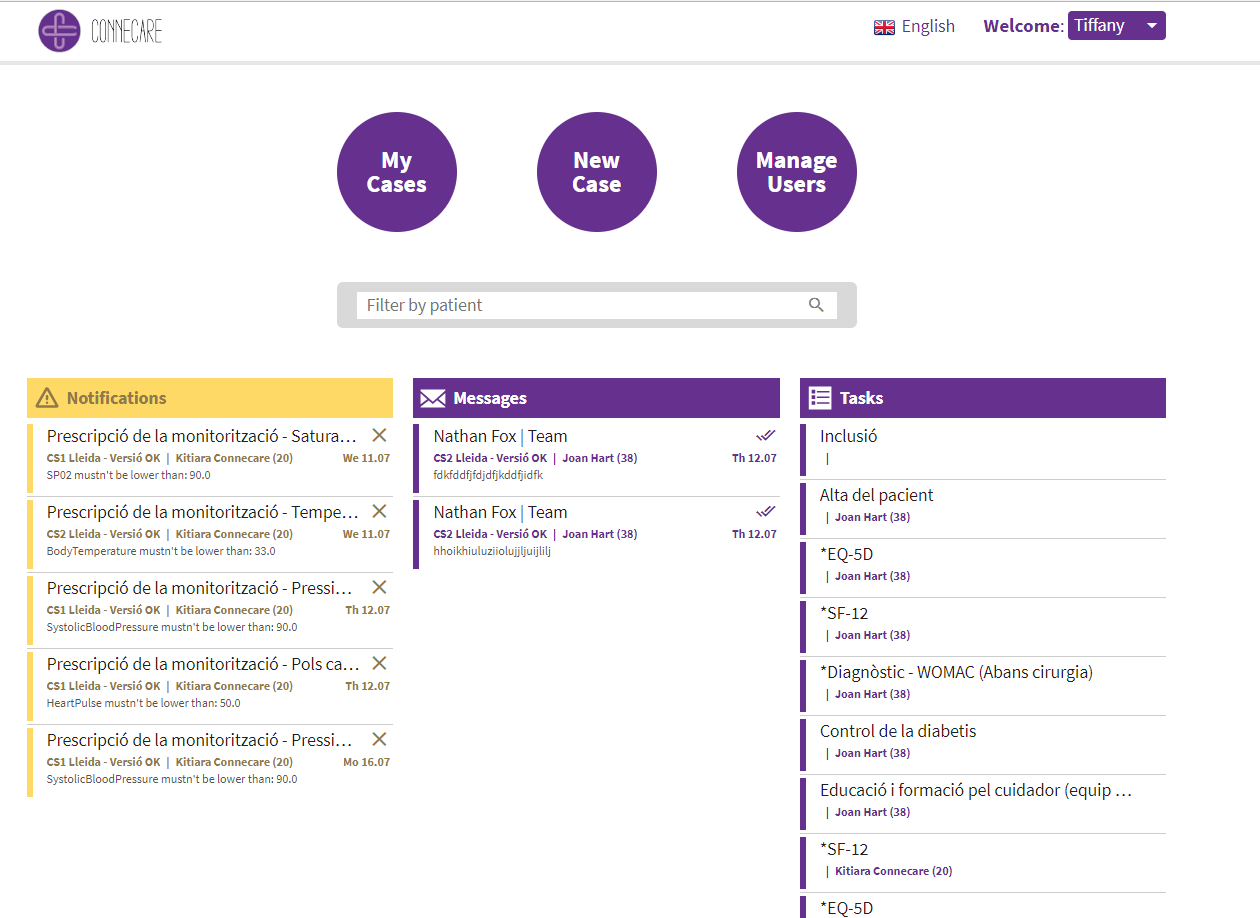


**Figure S1. Sample screenshot of the main screen of the SACM platform for the Case manager.**

**
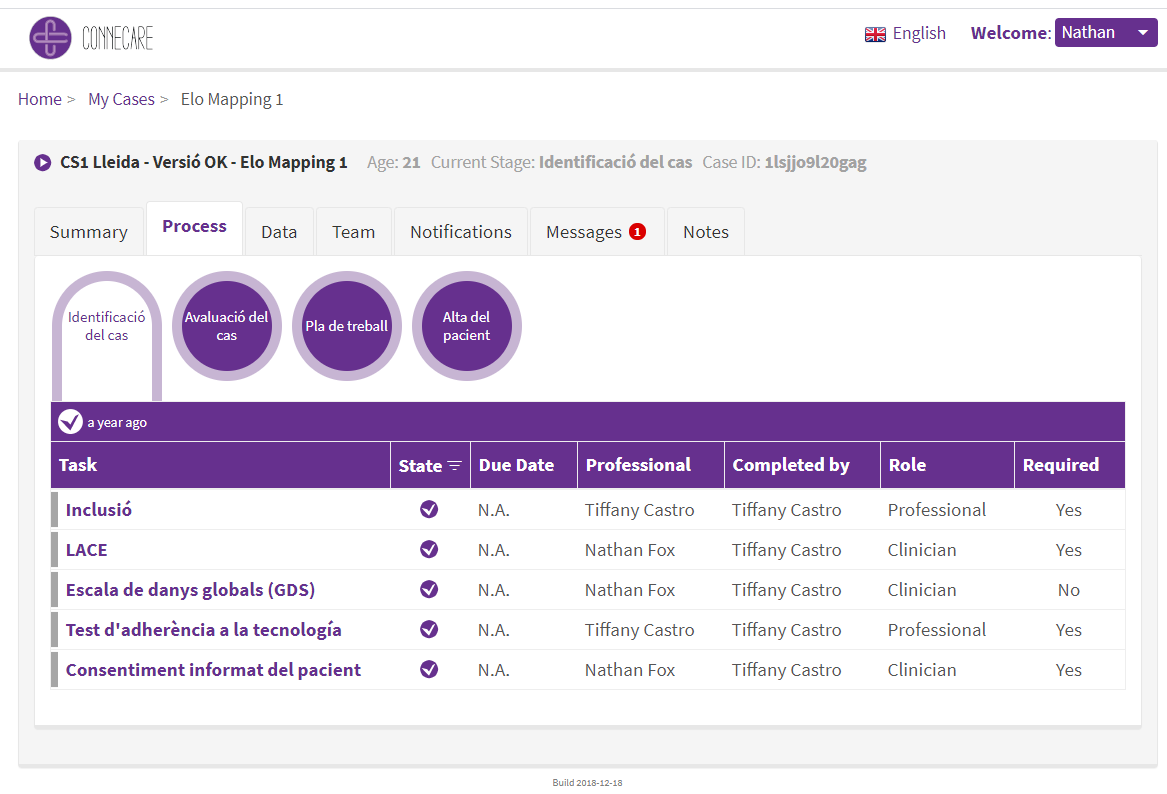
**

**Figure S2. Sample screenshot of the SACM platform, showing how the platform guides the case-manager through each step of case the identification.**

2. Case Evaluation

Once a patient was successfully included in the system, the first step consisted in assessing its baseline characteristics and performing the patient risk stratification based on clinical, environmental and social risk assessments. To this end, a set of questionnaires and tools implemented into the SACM were used (Table S2). This included tools assessing the characteristics of the patient and his environment: Functional Status and Autonomy (Barthel index for Activities of Daily Living); Comorbidities (Charlson index of comorbidities); Quality of life (SF-12); fall risk (Downton Fall Risk Index); mood (Hospital Anxiety and Depression (HAD) scale); mental status (Pfeiffer mental status questionnaire); nutritional status (body mass index); questions on communication and vision, characteristics of the patient’s dwelling, use of drugs, sleep habits, and use of tobacco and alcohol; and previous use of health resources. These assessments were performed by the nurse case-manager using a tablet computer. Once the information was collected (figure S3), the SACM automatically generated a summary screen for the patient (figure S4).


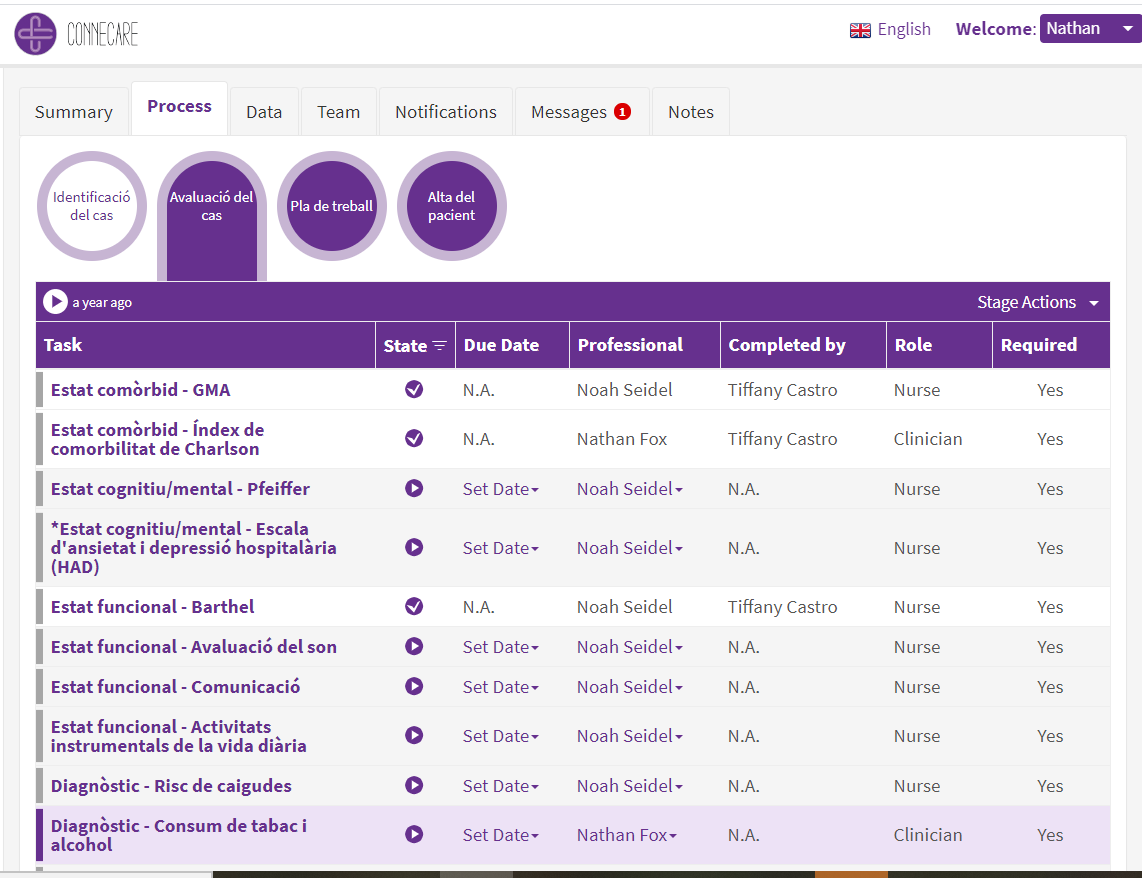


**Figure S3. Sample screenshot of the case evaluation process in the SACM platform.**


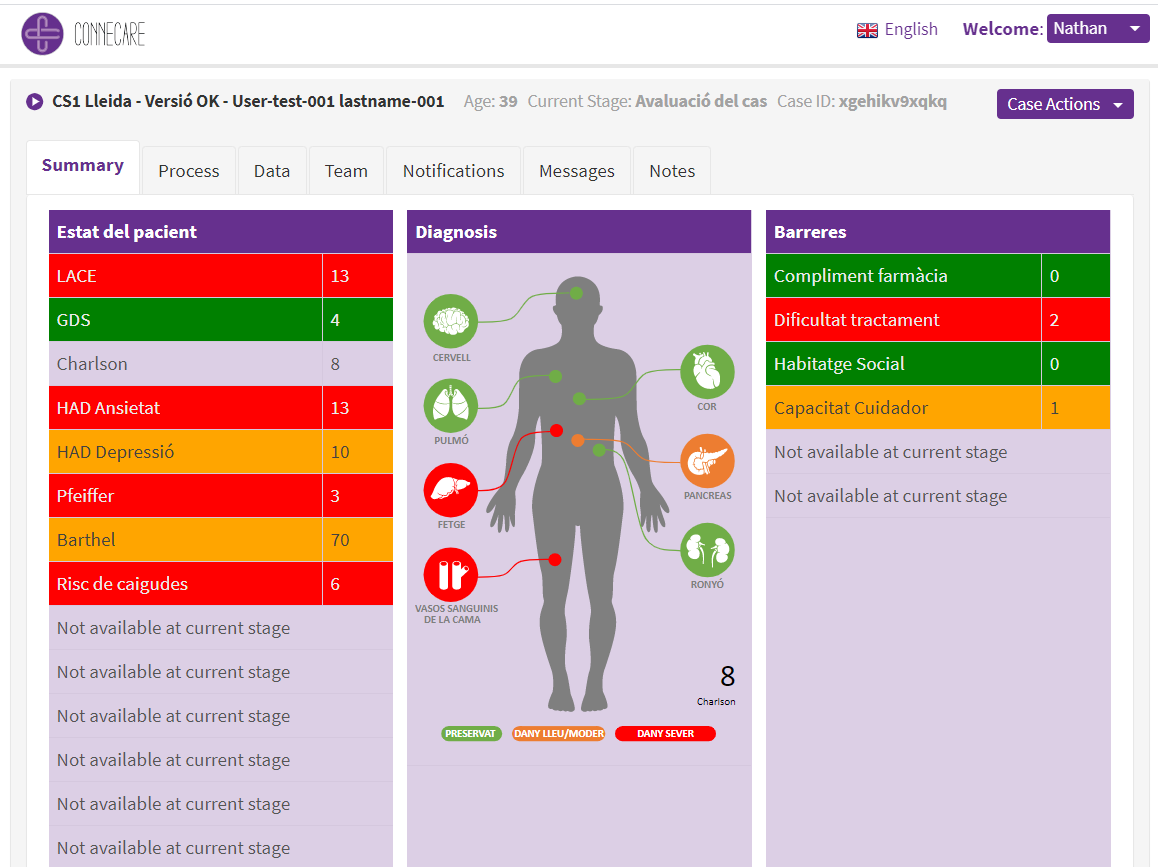


**Figure S4. Sample screenshot of the patient summary screen in the SACM platform.**

3. Work-plan definition

After the case evaluation, the case manager contacted the hospital physician who would define the main therapeutic strategies for the patient in agreement with the primary care team, which manages the patient in a day-to-day basis, and with the participation of the patient. A set of personalized proactive and preventive interventions would be agreed by all actors, and feed into the SACM platform. These included: a self-management app, with status and performance reports, a virtual coach with customizable automated feedback, full communication with the care team, and educational resources; a Fitbit Flex 2 digital activity tracker and any additional sensor deemed necessary by the care team (digital pulse-oximeter; digital scale; and, digital blood pressure (BP) monitor), all of them fully integrated into the self-management app; and any required drug treatment deemed necessary by the care team. Figure S5 shows sample screenshots showing some of the functionalities of the self-management app.


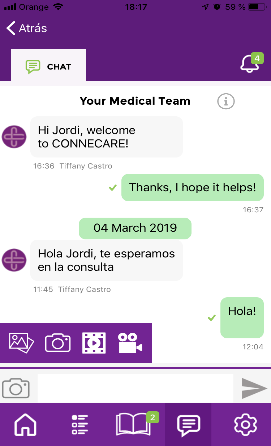

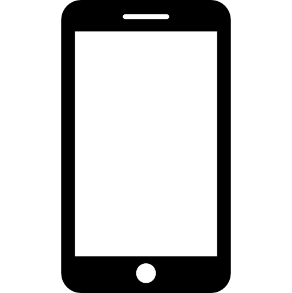

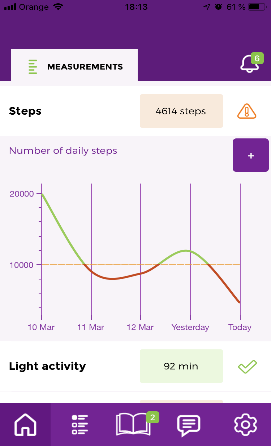

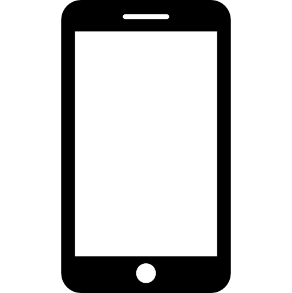

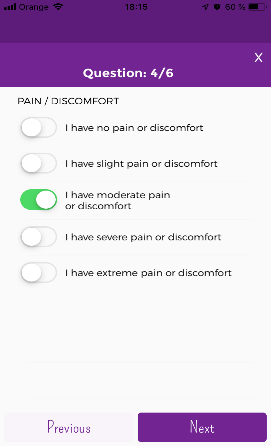

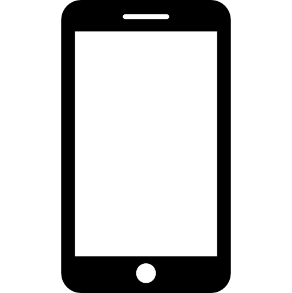

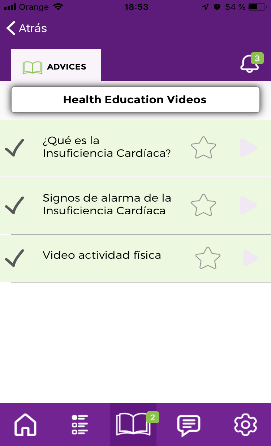

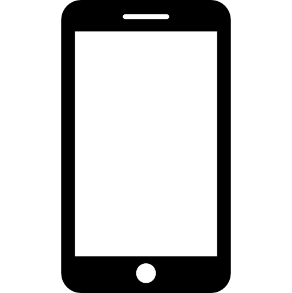


**Figure S5. Sample screenshots of the patient’s self-management app showing the administration of questionnaires, feedback on physical activity, communications with the care team, and educational resources.**

4. Work-plan execution

Once the work-plan was stablished, the progress of the patient was monitored throughout the work-plan execution phase. In this phase, the evolution of the personalized care plan was continuously monitored (through questionnaires, measurements with medical devices, educative material, and bidirectional communication wrapped-up in a patient self-management App). The patient’s self-management app, together with prescribed monitoring devices, was the key tool for self-monitoring the progress in the prescribed tasks as well as communicating with the care team (figure S5). The SACM was accessed by the members of the care team to check on the evolution of the patient and modify the therapeutic goals and/or tasks assigned to the patient. Moreover, the case manager overviewed the whole process and managed any alarms or signs, communicating with the patient and/or other members of the care team when needed. To this end, several summary screens were available to the case-manager in order to facilitate the simultaneous monitoring of all enrolled patients under its supervision (Figure S1 and S6). Among them, the geographical representation of patients in a map (mapping), combined with a traffic light system and multiple filtering and sorting options was very welcomed by participating professionals (Figure S6).


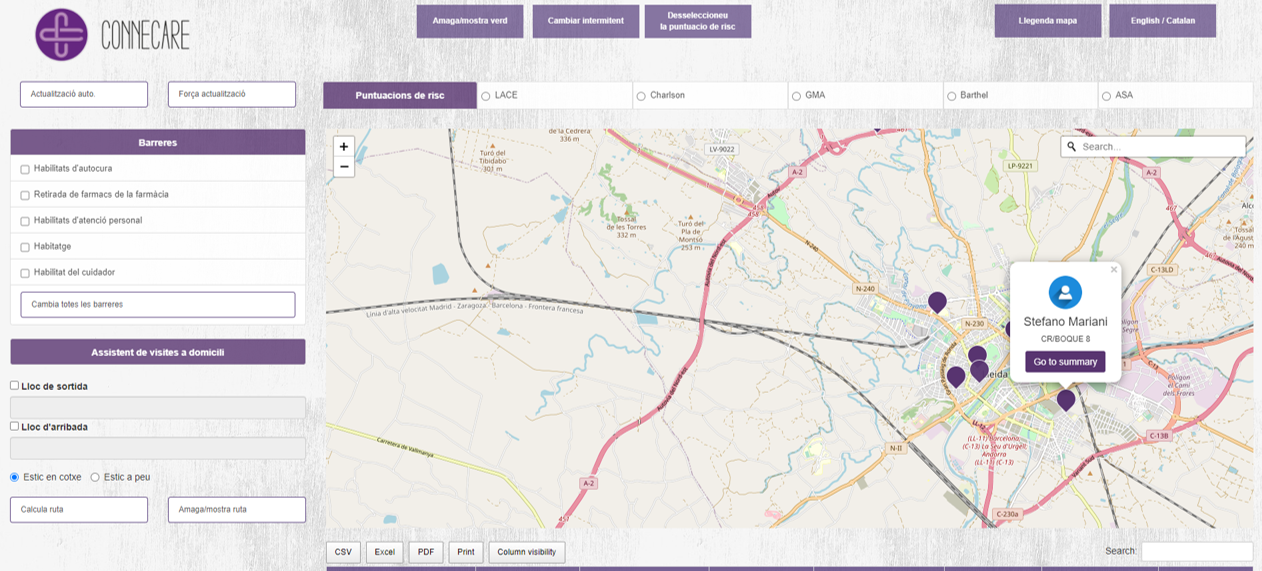


**Figure S6. Sample screenshot of the mapping functionally within the SACM.**

5. Discharge

The last step of the patient’s journey through the IC intervention was the discharge from the program, where the intervention was evaluated and all required feedback was collected. This task was performed by the case-manager and the hospital physician in care of the patient. It consisted on a visit where the patient and the professionals exposed their thoughts on the concluded IC program, the goals achieved, and the transfer of the management to the standard primary care management. The visit included the administration of several questionnaires and the collection of data from the EMR (Table S2).

# Technical structure of the supporting eHealth platform

The CONNECARE eHealth platform is a federation of subsystems each devoted to provide a set of goal-oriented functionalities, whose main components are the SMS app and the web-based SACM. Based on the concept of micro-services, the SMS provides intelligent tools to monitor patients (i.e., physical activity, sleeping, health status, drug adherence, simple rehabilitation tasks, and self-checked questionnaires) and to autonomously interact with them through engagement, rewards, and warnings through a recommender system. The SACM has extended functionalities for case modelling and execution, specifically tailored to the healthcare domain. Additionally, the SACM includes a Decision Support System to show patients in a map and to create routes for better organizing visits. The SMS and SACM interact each other through the CONNECARE Queue Manager, which connects both subsystems, orchestrates their communication, and provides an integration framework to link CONNECARE services to specific Electronic Health Records (EHR) and regional Personal Health Folders (PHF). Figure S7 sketches the architecture of the final CONNECARE system.


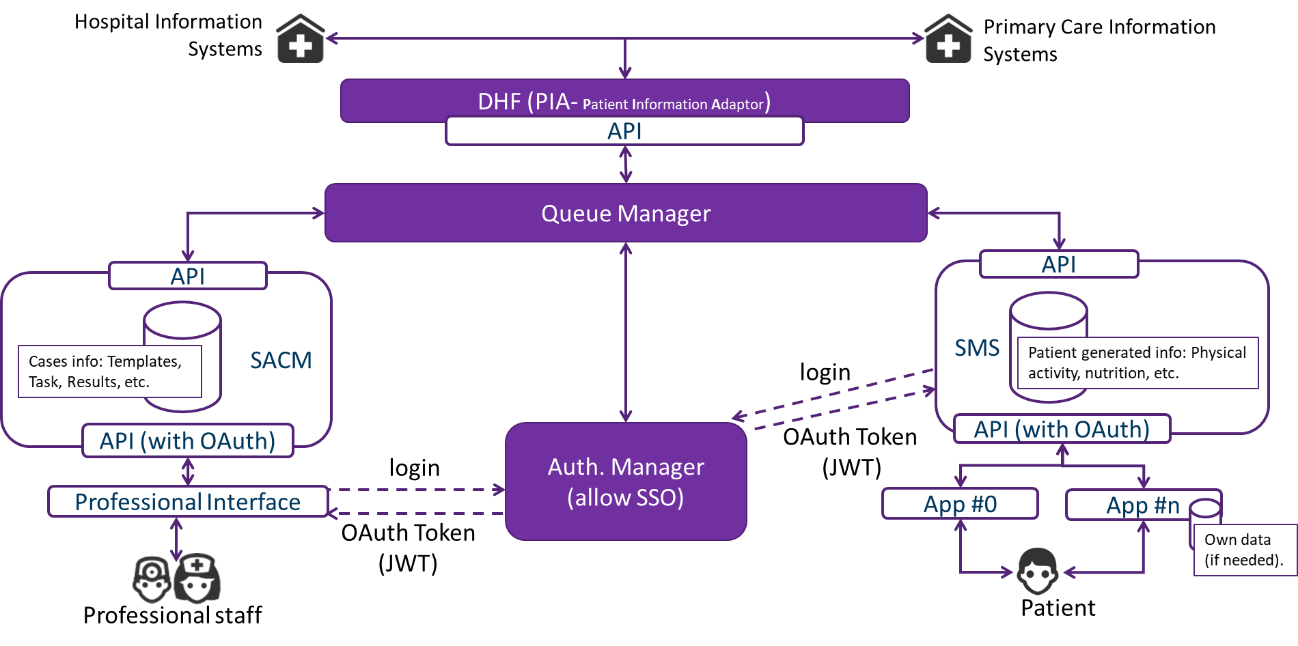


**Figure S7. Architecture of the CONNECARE eHealth system, including the web-based Smart Adaptive Case Management system (SACM) and the Self-Management System app (SMS).**

# Implementation efforts

On the one hand, the implementation of the IC model in Lleida required, first, the adaptation of the SACM platform. The self-management app needed to be fitted to the requirements and needs of the elderly CCP patients in Lleida. The SACM platform, needed to be the hub were all the professionals from different settings involved in the management of a given patient could exchange information, agree on the best management plan for each patient, and take specific actions in terms of treatment, monitoring and reactions when needed. Among others, the adaptations included the translation into Catalan and Spanish of all the self-management app and SACM platform contents, as well as a preliminary partial integration with legacy EMR in each setting (Argos SAP® (www.sap.com) and ECAP [1]). On the other hand, the implementation of the IC model required engaging a broad range of professionals and providing them with training and a fully functional access to the IC platform. Moreover, this required the emergence of new roles in the organization of the involved services. The already existing hospital case managers (that used to track a few proportion of patients), needed to be reinforced with a specific case-manager that took the role of both introducing the patient to the IC platform, and following-up the monitoring of the patients done by the involved health professionals.

# Costs estimations

The estimation of the costs of the IC program included the costs of newly required medical personnel, and the costs of licensing and running the IC platform. In the one hand, the IC model requires the incorporation of hospital-based nurse case-managers that are estimated to manage up to 500 simultaneous patients each. The cost of each case-manager is estimated to be 3500€ /month, thus resulting in a cost of 7€ /patient and month. It is assumed that the re-structuration of other staff’s time to include new tasks would be fully assumed by the health system and no additional personnel would be required, thus no additional cost would be generated. On the other hand, the cost of licensing and running the IC platform as well as the costs of maintaining, evolving and supporting it was obtained by analogy to costs of other health services like Home-based oxygen therapy, where a supplier covers the role of providing devices, licenses and technical support. This approach generated an estimated total cost of 16.67€ /patient and month. Overall, the direct costs of the IC program were estimated to be 23.67€ /patient and month. Therefore, the total cost of the 3-month IC program for the purpose of the current analyses was considered to be 71.01€ /patient and month. Finally, according to the official data provided by the Catalan Health Department (CVE-DOGC-A-13051031-2013) [2], the overall cost of unplanned medical visits in the health region of Lleida was 62€ and the cost of hospital admissions 555€ /day. No other direct or indirect costs were considered in the current analyses.

# References

1. ECAP. [http://salutweb.gencat.cat/ca/ambits_actuacio/linies_dactuacio
/tecnologies_informacio_i_comunicacio/ecap/](http://salutweb.gencat.cat/ca/ambits_actuacio/linies_dactuacio/tecnologies_informacio_i_comunicacio/ecap/) (last accessed 25/08/2020).

2. CVE-DOGC-A-13051031-2013: Resolution SLT/353/2013, of February 13, on the review of public prices for the services provided by the Catalan Health Institute. <https://www.comb.cat/Upload/Documents/4635.pdf> (last accessed 25/08/2020). [Catalan]

# Supplementary tables

| **Table S1. Within trial costs (average cost per patient) and cost-effectiveness, considering unplanned visits and hospital admissions related to the patient's main chronic diseases, in three Integrated Care (IC) program cost scenarios.** | | | | |
| --- | --- | --- | --- | --- |
|  | Usual Care | IC |  |  |
|  | (n=28) | (n=48) | Difference | ICER |
| Unplanned visits ^a^ | 56.69 | 24.80 | -31.89 |  |
| Hospital admissions ^a^ | 2267.57 | 1043.40 | -1224.17 |  |
| TOTAL medical costs per patient | 2324.26 | 1068.20 | -1256.06 |  |
|  | 1^st^ scenario (100% IC program costs) | | | |
| CONNECARE program | 0 | 71.01 | 71.01 |  |
| TOTAL costs per patient | 2324.26 | 1139.21 | -1185.05 | -237.96 |
|  | 2^nd^ scenario (150% IC program costs) | | | |
| CONNECARE program | 0 | 106.52 | 106.52 |  |
| TOTAL costs per patient | 2324.26 | 1174.72 | -1149.54 | -230.83 |
|  | 3^rd^ scenario (200% IC program costs) | | | |
| CONNECARE program | 0 | 142.02 | 142.02 |  |
| TOTAL costs per patient | 2324.26 | 1210.22 | -1114.04 | -223.70 |
| All costs expressed in Euros (€).  ^a^ Costs based on the Catalan Institute of Health official pricing (CVE-DOGC-A-13051031-2013).  IC: Integrated care; ICER: incremental cost-effectiveness ratio, Incremental cost associated with 1 additional point gain in SF12 | | | | |

**Table S2. Summary of the measurement tools used during the study.**
